# Molecular profiling of advanced solid tumors and patient outcomes with genotype-matched clinical trials: the Princess Margaret IMPACT/COMPACT trial

**DOI:** 10.1186/s13073-016-0364-2

**Published:** 2016-10-25

**Authors:** Tracy L. Stockley, Amit M. Oza, Hal K. Berman, Natasha B. Leighl, Jennifer J. Knox, Frances A. Shepherd, Eric X. Chen, Monika K. Krzyzanowska, Neesha Dhani, Anthony M. Joshua, Ming-Sound Tsao, Stefano Serra, Blaise Clarke, Michael H. Roehrl, Tong Zhang, Mahadeo A. Sukhai, Nadia Califaretti, Mateya Trinkaus, Patricia Shaw, Theodorus van der Kwast, Lisa Wang, Carl Virtanen, Raymond H. Kim, Albiruni R. A. Razak, Aaron R. Hansen, Celeste Yu, Trevor J. Pugh, Suzanne Kamel-Reid, Lillian L. Siu, Philippe L. Bedard

**Affiliations:** 1 Laboratory Medicine Program, University Health Network, Toronto, Canada; 2 Department of Laboratory Medicine and Pathobiology, University of Toronto, Toronto, Canada; 3 Cancer Genomics Program, Princess Margaret Cancer Centre, Toronto, Canada; 4 Division of Medical Oncology and Hematology, Princess Margaret Cancer Centre, 610 University Avenue, Toronto, M5G 2M9 Canada; 5 Department of Medicine, University of Toronto, Toronto, Canada; 6 Department of Medical Biophysics, University of Toronto, Toronto, Canada; 7 Department of Oncology, Grand River Regional Cancer Centre, Kitchener-Waterloo, Canada; 8 Department of Oncology, McMaster University, Faculty of Health Sciences, Hamilton, Canada; 9 Department of Medicine, Markham Stouffville Hospital, Markham, Canada; 10 Department of Biostatistics, Princess Margaret Cancer Centre, Toronto, Canada; 11 Princess Margaret Research Institute, Princess Margaret Cancer Centre, Toronto, Canada

**Keywords:** Molecular profiling, DNA sequencing, Clinical trials, Solid tumors, Precision medicine

## Abstract

**Background:**

The clinical utility of molecular profiling of tumor tissue to guide treatment of patients with advanced solid tumors is unknown. Our objectives were to evaluate the frequency of genomic alterations, clinical “actionability” of somatic variants, enrollment in mutation-targeted or other clinical trials, and outcome of molecular profiling for advanced solid tumor patients at the Princess Margaret Cancer Centre (PM).

**Methods:**

Patients with advanced solid tumors aged ≥18 years, good performance status, and archival tumor tissue available were prospectively consented. DNA from archival formalin-fixed paraffin-embedded tumor tissue was tested using a MALDI-TOF MS hotspot panel or a targeted next generation sequencing (NGS) panel. Somatic variants were classified according to clinical actionability and an annotated report included in the electronic medical record. Oncologists were provided with summary tables of their patients’ molecular profiling results and available mutation-specific clinical trials. Enrolment in genotype-matched versus genotype-unmatched clinical trials following release of profiling results and response by RECIST v1.1 criteria were evaluated.

**Results:**

From March 2012 to July 2014, 1893 patients were enrolled and 1640 tested. After a median follow-up of 18 months, 245 patients (15 %) who were tested were subsequently treated on 277 therapeutic clinical trials, including 84 patients (5 %) on 89 genotype-matched trials. The overall response rate was higher in patients treated on genotype-matched trials (19 %) compared with genotype-unmatched trials (9 %; *p* < 0.026). In a multi-variable model, trial matching by genotype (*p* = 0.021) and female gender (*p* = 0.034) were the only factors associated with increased likelihood of treatment response.

**Conclusions:**

Few advanced solid tumor patients enrolled in a prospective institutional molecular profiling trial were treated subsequently on genotype-matched therapeutic trials. In this non-randomized comparison, genotype-enrichment of early phase clinical trials was associated with an increased objective tumor response rate.

**Trial registration:**

NCT01505400 (date of registration 4 January 2012).

**Electronic supplementary material:**

The online version of this article (doi:10.1186/s13073-016-0364-2) contains supplementary material, which is available to authorized users.

## Background

Molecular profiling can provide diagnostic, prognostic, or treatment-related information to guide cancer patient management. Advances in next-generation sequencing (NGS) have enabled multiplex testing to overcome the constraints associated with sequential single-analyte testing [1–3]. Large-scale research projects have elucidated genomic landscapes of many cancers but have provided limited insight into the clinical utility of genomic testing. Our aim was to evaluate if targeted DNA profiling improves outcomes for patients assigned to clinical trials based on knowledge of actionable somatic mutations.

At the Princess Margaret Cancer Centre (PM), the Integrated Molecular Profiling in Advanced Cancers Trial (IMPACT) and Community Molecular Profiling in Advanced Cancers Trial (COMPACT) are prospective studies that provide molecular characterization data to oncologists to match patients with advanced solid tumors to clinical trials with targeted therapies. Here, we report the frequency of alterations, clinical “actionability” of the somatic variants, clinical trial enrollment, and outcome based upon molecular profiling results.

## Methods

### Patient cohort

For IMPACT, patients with advanced solid tumors treated at PM were prospectively consented for molecular profiling during a routine clinical visit. For COMPACT, patients with advanced solid tumors treated at other hospitals in Ontario were referred to a dedicated weekly clinic at PM for eligibility review, consent, and blood sample collection. Eligible patients had advanced solid tumors, were aged ≥18 years, had Eastern Cooperative Oncology Group (ECOG) performance status ≤1, and had available formalin-fixed paraffin-embedded (FFPE) archival tumor tissue. The University Health Network Research Ethics Board approved this study (#11-0962-CE). Enrollment for IMPACT began on 1 March 2012 and for COMPACT on 16 November 2012 and ended on 31 July 2014 for this analysis.

### Specimens

DNA was extracted from sections of FFPE tumor specimens from biopsies or surgical resections. If multiple archival tumor specimens were available, the most recent archival FFPE specimen was reviewed, with a minimum acceptable tumor cellularity of 10 %. Tumor regions were isolated by 1–2 × 1 mm punch from FFPE blocks or manual macrodissection of unstained material from 15–20 slides. FFPE samples were deparaffinized, cells lysed with proteinase K, and DNA extracted using the QIAmp DNA FFPE Tissue Kit (Qiagen, Germantown, MD, USA). DNA was quantified using the Qubit dsDNA Assay kit on the Qubit 2.0 Fluorometer (ThermoFisher Scientific, Waltham, MA, USA).

Participants provided a peripheral blood sample (5 mL in EDTA-coated tubes) as a source of matched germline DNA. DNA was extracted using either standard manual phenol/chloroform extraction methods or automated extraction (MagAttract DNA Mini M48 kit; Qiagen). Patients were offered return of pathogenic germline results at the time of consent and asked to identify a family member delegate who could receive results on their behalf if required.

### Molecular profiling assays

All testing was performed in a laboratory accredited by the College of American Pathologists (CAP) and certified to meet Clinical Laboratory Improvement Amendments (CLIA). Three molecular profiling assays were used over the study period: a custom multiplex genotyping panel on a matrix-assisted laser desorption/ionization time-of-flight (MALDI-TOF) mass-spectrometry platform (MassARRAY, Agena Bioscience, San Diego, CA, USA) to genotype 279 mutations within 23 genes (Additional file 1: Table S1); the TruSeq Amplicon Cancer Panel (TSACP, Illumina) on the MiSeq sequencer (Illumina) covering regions of 48 genes (Additional file 1: Table S2); and the Ion AmpliSeq Cancer Panel (ASCP, ThermoFisher Scientific) on the Ion Proton sequencer (ThermoFisher Scientific) covering regions of 50 genes (Additional file 1: Table S3). For more in-depth methodology on molecular profiling assays, including sequence alignment and base calling, see Additional file 1: Supplementary Methods.

### Variant assessment and classification

Variants were assessed and classified according to the classification scheme of Sukhai et al. [4]. Briefly, a five-class scheme was used to sort variants according to actionability (defined as providing information on prognosis, prediction, diagnosis, or treatment), recurrence of variants in specific tumor sites, and known or predicted deleterious effects on protein function. Interpretation and data integration were performed using Alamut v.2.4.5 (Interactive Biosoftware, Rouen, France). Primary review, assessment, and classification of all variants were independently performed by a minimum of two assessors followed by a third review prior to reporting, with cases where assessors disagreed resolved by group discussion.

### Immunohistochemistry (IHC)

Phosphatase and tensin homolog (PTEN) IHC was performed using rabbit monoclonal Ab 138G6 (Cell Signaling Technology, Danvers, MA, USA) on a Dako platform using a dilution of 1:50 and Flex + 30 protocol. Complete absence of tumor cell staining with positive staining of surrounding tumor stroma fibroblasts/endothelial cells was used to denote PTEN deficiency [5].

### Return of testing results

The molecular profiling report was included in the electronic medical record and returned to the treating oncologist. The clinical significance of profiling results was discussed with PM patients during a routine clinic visit by their treating oncologist. A PM oncologist reviewed results with patients treated at other hospitals by telephone. All oncologists were provided with regular summary tables of testing results and mutation-specific clinical trial listings available at PM. A monthly genomic tumor board was convened at PM to establish consensus treatment recommendations for patients with complex profiling results. A committee consisting of a molecular geneticist, medical geneticist, genetic councilor, and medical oncologist reviewed pathogenic germline variants before return of germline testing results. Germline results were disclosed to the patient or designate by a genetic counselor or medical geneticist.

### Clinical data collection

For each patient, baseline patient and tumor characteristics, treatment regimen(s), time on treatment(s) and survival were retrieved from medical records and updated every three months. Therapeutic clinical trial enrollment was evaluated from the date of reporting molecular profiling results until 9 January 2015. Genotype-matched trials were defined as studies with eligibility criteria restricted to patients with specific somatic mutations, those with a targeted drug with enriched clinical or preclinical activity in a patient’s genotype, or those with a drug that inhibited a pathway directly linked to the somatic mutation. Decisions about trial enrollment were based upon trial availability, patient or physician preference, and did not follow a pre-specified algorithm. Targeted lesion measurements and RECIST 1.1 [6] assessments were performed by radiologists.

### Statistics

Descriptive statistics were used to summarize patient characteristics, profiling results, and anti-tumor activity. Comparisons between patients with profiling results treated on genotype-matched and genotype-unmatched trials were performed using a generalized estimating equation (GEE) model [7]. A multi-variable GEE model for response included trial matching by genotype, gender, trial phase, number of lines of prior systemic therapy, investigational agent class, age, tumor type, and sequencing platform. A mixed model was used to compare time on treatment, defined as the date of trial enrollment until the date of discontinuation of investigational treatment. A robust score test was used to compare overall survival following trial enrolment between genotype-matched and genotype-unmatched groups [8]. These comparisons accounted for individual patients who were included on multiple therapeutic trials [8]. Differences with *p* values of < 0.05 were considered statistically significant.

## Results

### Patient cohort

A total of 1893 patients were enrolled, including gynecological (23 %), breast (18 %), lung (18 %), colorectal (17 %), pancreatobiliary (8 %), upper aerodigestive (6 %), genitourinary (5 %), and other (5 %) cancers (Table 1). The median age was 59 years (age range, 18–89 years); patients were predominantly female (69 %); had received a median of 2 prior systemic treatments (range, 1–18), and had excellent performance status (43 % PS0 and 56 % PS1). Of 253 (13 %) screen failures, 10 % were for insufficient tissue or DNA and 3 % for clinical deterioration or other reasons. The median follow-up from reporting results was 18 months (range, 1–33 months). A total of 651 (40 %) patients were deceased at the time of the database lock.

**Table 1:** Characteristics of patients enrolled into IMPACT/COMPACT (n = 1893)

Patients enrolled	Patients enrolled	Patients profiled	Patients profiled enrolled on any therapeutic trials	Patients profiled enrolled on genotype-matched trials
Median age (range)	59 (18–89)	58 (18–89)	58 (18–81)	58 (24–81)
Female/Male	1303/590 (59 %)/(31 %)	1166/479 (71 %)/(29 %)	205/72 (74 %)/(26 %)	64/25 (72 %)/(28 %)
Median lines of prior treatment (range)	2 (1–18)	2 (1–18)	2 (1–16)	2 (1–11)
ECOG performance status (0/1/2)	43 %/56 %/<1 %	44 %/55 %/<1 %	78 %/22 %/0 %	78 %/22 %/0 %
Median time from collection of archival tumor sample to profiling report in years (range)		1.6 (0.1-24.9)	1.7 (0.1-18.9)	1.9 (0.1-18.9)
Primary lesion/Metastatic lesion profiled		1080/560	193/84	66/23
		(66 %)/(34 %)	(70 %)/(30 %)	(74 %)/(26 %)
Tumor types				
Breast	341 (18 %)	310 (19 %)	41/310 (13 %)	19/310 (6 %)
Colorectal	326 (17 %)	299 (18 %)	38/299 (13 %)	18/299 (6 %)
Gynecological	430 (23 %)	405 (25 %)	80/405 (20 %)	20/405 (5 %)
Lung	339 (18 %)	256 (16 %)	43/256 (17 %)	18/256 (7 %)
Genitourinary	92 (5 %)	74 (5 %)	9/74 (12 %)	4/74 (5 %)
Pancreatobiliary	151 (8 %)	104 (6 %)	9/104 (9 %)	1/104 (1 %)
Upper aerodigestive	115 (6 %)	102 (6 %)	8/102 (8 %)	2/102 (2 %)
Other	99 (5 %)	81 (5 %)	17/81 (21 %)	2/81 (2 %)
TOTAL	1893	1640	245/1640 (15 %)	84/1640 (5 %)

### Molecular profiling

**Fig. 1:**
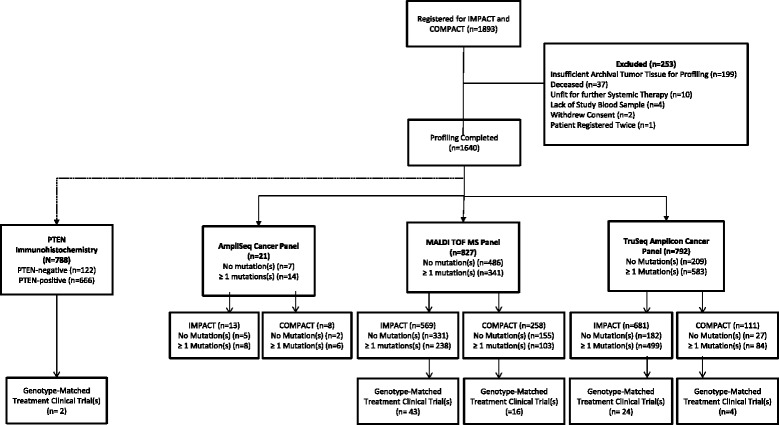
CONSORT diagram

**Fig. 2:**
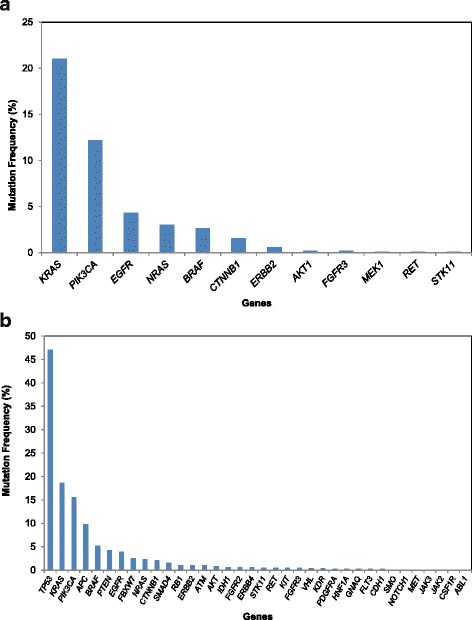
Mutation frequency by gene from results of (**a**) MALDI-TOF, n = 827, and (**b**) TruSeq Amplicon Cancer Panel, n = 792. Mutation frequency was calculated as number of variant occurrences within each gene divided by the total number of patients

Successful molecular profiling was achieved in 1640 patients (87 %), 827 (50 %) had samples tested by MALDI-TOF MS, 792 (48 %) by TSACP, and 21 (1 %) by ASCP (Fig. 1). One or more somatic mutations were detected in 341 (41 %) patients tested by MALDI-TOF MS, 583 (74 %) by TSACP, and 14 (67 %) by ASCP. Median laboratory turnaround time (sample receipt to report) was 32 days (range, 6–228 days). Of patient samples tested by MALDI-TOF MS, *KRAS* (21 %) was the most frequently mutated gene, followed by *PIK3CA* (12 %), with additional genes in the range of 1–5 % frequency. Of samples tested by the TSACP, *TP53* had the highest mutation frequency (47 % of all identified variants), followed by *KRAS, PIK3CA*, and *APC* with mutation frequencies in the range of 5–15 % and the remainder <5 % (Fig. 2). We attribute the difference in mutation landscape between these two platforms to inclusion of *TP53* in the TSACP assay but not in MALDI-TOF (see Additional file 1: Supplemental Methods).

**Fig. 3:**
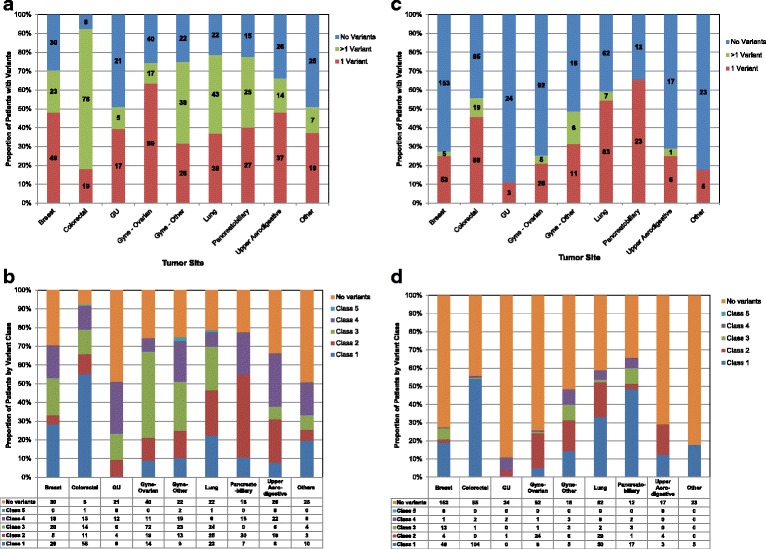
Distribution of patients by tumor site and most actionable variant identified [4]. Cases tested with TruSeq Amplicon Cancer Panel (TSACP; n = 792) are shown in (**a**) and (**b**); cases tested by MALDI-TOF MS (n = 827) are shown in (**c**) and (**d**). **a** Proportion and number of variants by tumor site, TSACP. **b** Actionability of variants by tumor site, TSACP. **c** Proportion and number of variants by tumor site, MALDI-TOF. **d** Actionability of variants per case by tumor site, MALDI-TOF. Patients with more than one variant were counted once by their most actionable variant class. Total number of patients is indicated by value within or below each *bar section*. “Gyne-other” includes cervical, endometrial, fallopian tube, uterine, and vulvar

Class 1 and 2 variants are the most clinically significant with known actionability for the specific variant in the tumor site tested (Class 1) or a different tumor site (Class 2) [4]. More than 20 % of patients with breast, colorectal, gynecologial, lung, or pancreatobiliary cancers had Class 1 or 2 variants detected by TSACP or MALDI-TOF (Fig. 3). Of patients with genitourinary cancers, only 9 % had actionable variants identified on TSACP and 3 % on MALDI-TOF. For patients with other solid tumors, 25 % had actionable variants identified on TSACP and 18 % on MALDI-TOF. PTEN protein expression was lost by IHC for 122/788 (15 %) tumors tested. *PTEN* gene mutations were detected by NGS in 14/122 (11.5 %) tumors that were PTEN-negative by IHC.

### Clinical trials and outcomes

Of the 1640 patients with molecular profiling results, 245 (15 %) were subsequently enrolled in 277 therapeutic clinical trials, including 84 (5 %) treated on 89 genotype-matched trials (Table 2). Patients with pancreatobiliary, upper aerodigestive tract, and other solid tumors were least likely to be treated on genotype-matched trials. Somatic mutations in four genes (*PIK3CA*, *KRAS*, *BRAF*, and *EGFR*) accounted for 76/89 (85 %) of genotype-matched trial enrollments: including *PIK3CA* for breast cancer (20/22); *BRAF* (5/18) and *KRAS* (9/18) for colorectal cancer; *KRAS* (9/18) and *EGFR* (7/18) for non-small cell lung cancer; and *KRAS* (14/22) and *PIK3CA* (7/22) for gynecological cancers. A complete list of genotype-matched clinical trials by drug class, somatic genotype (variant level), and tumor type are summarized in Table 3.

**Table 2:** Characteristics of patients enrolled in therapeutic trials following molecular profiling

	All trials	Genotype-matched	Genotype-unmatched	*p* value
Median age (range)	58 (18–81)	58 (24–81)	58.5 (18–80)	NS
Female/Male	205/72	64/25	141/47	NS
Median prior systemic therapies (range)	2 (1–16)	2 (1–11)	2 (1–16)	NS
Tumor type (number of patients)				
Breast	47	22	25	NS
Colorectal	43	18	25	
Lung	48	18	30	
Gynecological	91	22	69	
Other	48	9	39	
Genotyping platform (number of patients)				
MALDI-TOF MS panel	176	61	115	NS
TruSeq Amplicon cancer panel	101	28	73	
Ampliseq cancer panel	0	0	0	
Trial phase (number of patients)				
Phase I	158	72	86	<0.001
Phase II	67	9	58	
Phase III	52	8	44	
Investigational agent(s) (number of patients)				
Targeted monotherapy	112	23	89	<0.001
Targeted drug combination	87	59	28	
Targeted drug and chemotherapy	43	7	36	
Immunotherapy	34	0	34	
Radiotherapy	1	0	1	

*NS* not significant

**Table 3:** Genotype-matched clinical trials by drug class, somatic genotypes (variant level), and tumor type (*n* = 89)

Tumor type	Somatic genotype (variant)	Genotype-matching trial drug class	Target lesion percent change
Colorectal	*No mutations (KRAS wildtype)*	EGFR	−100 %
Breast	*PIK3CA E545K*	PI3K, Endocrine	−90 %
Colorectal	*BRAF V600E*	BRAF, PI3K, EGFR	−85 %
Colorectal	*BRAF V600E*	BRAF, PI3K, EGFR	−81 %
	*TP53 S215G*		
Gynecological	*KRAS G13D*	PI3K, MEK	−70 %
Gynecological	*PIK3CA H1047R*	VEGF	−64 %
	*KRAS G12D*		
Gynecological	*KRAS G12V*	PI3K, MEK	−63 %
Breast	*PIK3CA H1047R*	PI3K, IGF1R	−61 %
Lung	*EGFR E746_A750del*	EGFR	−58 %
	*EGFR T790M*		
Lung	*KRAS G13D*	MEK	−54 %
Breast	*PIK3CA H1047R*	PI3K	−50 %
Lung	*EGFR L858R*	EGFR	−47 %
	*EGFR T790M*		
	*CTNNB1 S37C*		
Gynecological	*KRAS G12D*	MEK	−47 %
Gynecological	*KRAS G12V*	PI3K, IGF1R	−45 %
	*NRAS Q61R*		
Gynecological	*KRAS G12A*	PI3K, MEK	−38 %
Breast	*PIK3CA N345K*	ANG2, MTOR	−37 %
Lung	*KRAS G12D*	PI3K, MEK	−37 %
Gynecological	*PIK3CA H1047R*	PI3K, MEK	−37 %
	*KRAS G12D*		
Lung	*KRAS G12C*	MEK	−28 %
Gynecological	*TP53 K132N*	WEE1	−26 %
Gynecological	*KRAS G12D*	PI3K, MEK	−25 %
Colorectal	*BRAF V600E*	BRAF, PI3K, EGFR	−24 %
	*TP53 R273C*		
	*PIK3CA Q546K*		
	*APC E1544X*		
Breast	*ERBB2 D769H*	HER2	−23 %
	*PIK3CA N345K*		
Upper aerodigestive	*BRAF V600E*	MEK	−20 %
Lung	*KRAS G12S*	MEK	−20 %
Gynecological	*KRAS G12D*	MEK, PI3K	−20 %
Colorectal	*BRAF V600E*	BRAF, PI3K, EGFR	−20 %
	*TP53 R175H*		
	*TP53 Q165X*		
Breast	*FGFR2 Y376C*	FGFR	−19 %
Breast	*PIK3CA H1047L*	PI3K	−18 %
Other	*GNAQ Q209P*	MEK	−18 %
Lung	*EGFR L858R*	HER3, EGFR	−18 %
Lung	*KRAS G12A*	PI3K, MEK	−17 %
Lung	*EGFR L858R*	HER3, EGFR	−17 %
Colorectal	*KRAS G12D*	PI3K, MEK	−16 %
	*PIK3CA E545K*		
Gynecological	*KRAS G12V*	PI3K, MEK	−16 %
Gynecological	*BRAF V600E*	MEK	−15 %
Gynecological	*KRAS G12D*	PI3K, MEK	−15 %
Lung	*KRAS G12V*	PI3K, MEK	−13 %
Colorectal	*KRAS G12S*	MEK, EGFR	−13 %
Gynecological	*NRAS Q61K*	PI3K, MEK	−13 %
Pancreatobiliary	*KRAS G12V*	PI3K, IGF1R	−13 %
Gynecological	*PIK3CA H1047R*	ANG2, mTOR	−9 %
Breast	*PIK3CA E545K*	AKT	−7 %
Colorectal	*KRAS G12V*	MEK, EGFR	−7 %
Breast	*ERBB2 D769H*	PI3K, IGF1R	−7 %
	*PIK3CA N345K*		
Lung	*KRAS G12V*	MEK	−6 %
Colorectal	*ERBB2 L755S*	VEGF, ANG2	−6 %
	*BRAF N581S*		
	*ERBB2 L755S*		
	*APC Q1429fs*		
Genitourinary	*No mutations*	PI3K	−5 %
	*PTEN negative on IHC*		
Lung	*EGFR L858R*	EGFR	−4 %
Lung	*EGFR L858R*	EGFR	−4 %
Lung	*EGFR E746_A750del*	EGFR	−3 %
Breast	*PIK3CA H1047R*	AKT	−3 %
Upper aerodigestive	*KRAS G12V*	PI3K, MEK	−2 %
Gynecological	*KRAS G12D*	PI3K, MEK	−2 %
Colorectal	*KRAS G13D*	MEK, EGFR	−0.6 %
Genitourinary	*No mutations*	PI3K	0 %
	*PTEN negative on IHC*		
Breast	*PIK3CA H1047R*	PI3K	0 %
Colorectal	*PIK3CA E542K*	PI3K	+0.5 %
	*KRAS G12V*		
Colorectal	*No Mutations (KRAS wildtype)*	MEK, EGFR	+2 %
Colorectal	*KRAS G13D*	PI3K, MEK	+2 %
	*PIK3CA E545K*		
Breast	*PIK3CA H1047L*	PI3K, IGF1R	+4 %
Genitourinary	*No mutations*	PI3K	+4 %
	*PTEN negative on IHC*		
Gynecological	*PIK3CA E545K*	MTOR	+5 %
Breast	*PIK3CA N345K*	PI3K, IGF1R	+6 %
Breast	*PIK3CA N345K*	PI3K, MEK	+6 %
	*NRAS G12D*		
Gynecological	*PIK3CA C420R*	FGFR, PI3K	+8 %
Colorectal	*KRAS G12D*	MEK, EGFR	+9 %
Lung	*PIK3CA E545K*	PI3K, IGF1R	+11 %
Colorectal	*No mutations (KRAS wildtype)*	MEK, EGFR	+11 %
Colorectal	*KRAS G12D*	PI3K, MEK	+12 %
	*PIK3CA Q546K*		
Gynecological	*PIK3CA H1047R*	PI3K, IGF1R	+12 %
Breast	*PIK3CA p.Glu545Gly*	AKT	+28 %
	*PTEN p.Leu320X*		
	*PIK3CA p.Arg93Gln*		
Gynecological	*TP53 R175H*	PI3K	+29 %
	*PIK3CA R93W*		
	*FBXW7 R479Q*		
Colorectal	*KRAS G12D*	PI3K, MEK	+30 %
	*PIK3CA E545K*		
Genitourinary	*PIK3CA p.Asn345Lys*	PI3K	+31 %
Breast	*PIK3CA E545K*	FGFR, PI3K	+32 %
Breast	*PIK3CA H1047R*	PI3K, IGF1R	+39 %
Colorectal	*No mutations (KRAS wildtype)*	MEK, EGFR	+55 %
Breast	*PIK3CA E542K*	PI3K, Endocrine	+66 %
Breast	*PIK3CA N345K*	PI3K, IGF1R	NE
Lung	*KRAS G12C*	PI3K, MEK	NE
Breast	*PIK3CA H1047R*	PI3K, IGF1R	NE
Lung	*KRAS G12A*	MEK	NE
	*PIK3CA H1047R*		
Gynecological	*KRAS G12A*	PI3K, IGF1R	NE
Breast	*PIK3CA E545K*	PI3K	NE
	*TP53 L252del*		
	*BRAF c.1315-4C > G*		
Gynecological	*KRAS G12V*	MEK	NE
Lung	*BRAF V600E*	MEK	NE
Breast	*PIK3CA H1047L*	PI3K	NE
	*TP53 C238Y*		
Other	*KIT V559A*	PI3K, EGFR	NE

**Fig. 4:**
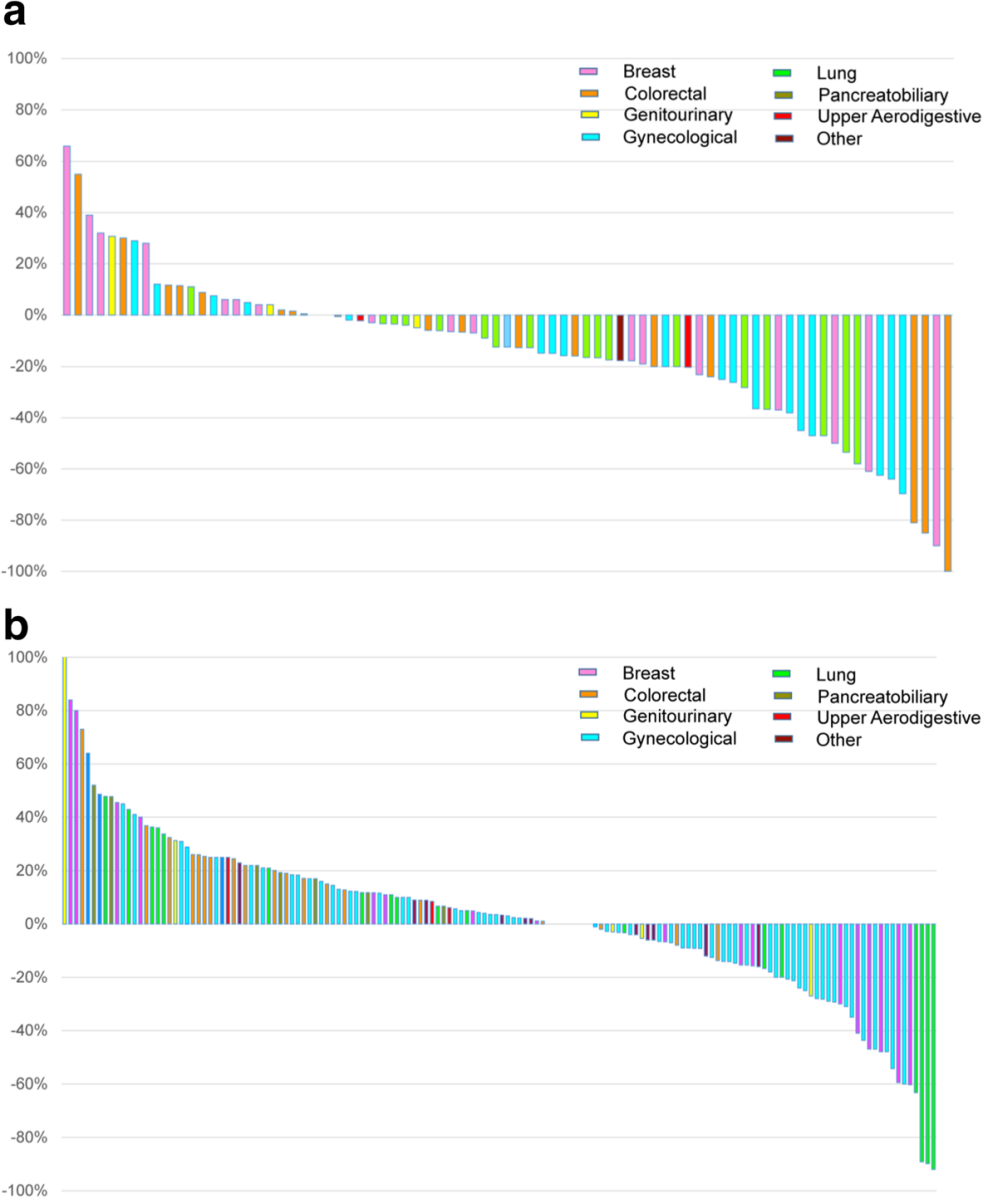
**a** *Waterfall plot* of best tumor shrinkage of target lesions by RECIST for patients treated on (**a**) genotype-matched clinical trials (n = 79) and (**b**) genotype-unmatched clinical trials (n = 150)

The age and sex distribution, as well as the number of lines of prior systemic therapy, were similar between the genotype-matched and genotype-unmatched trial patient cohorts (Table 2). There was no difference in the proportion of trials that were genotype-matched between patients profiled on MALDI-TOF MS (61/176 [35 %]) compared with TSACP (28/101 [28 %]; *p* = 0.24). A higher proportion of genotype-matched trial patients were treated in phase I studies (81 %) compared with genotype-unmatched trials (46 %; *p* < 0.001). Genotype-matched trial patients were more likely to be treated with targeted drug combinations without chemotherapy or immunotherapy. The overall response rate was higher in patients treated on genotype-matched trials (19 %) compared with genotype-unmatched trials (9 %; *p* = 0.026) (Fig. 4). In multi-variable analysis, trial matching according to genotype (*p* = 0.021) and female gender (*p* = 0.034) were the only statistically significant factors associated with response (Additional file 1: Table S4). Genotype-matched trial patients were more likely to achieve a best response of any shrinkage in the sum of their target lesions (62 %) compared with genotype-unmatched trial patients (32 %; *p* < 0.001). There was no difference in the time on treatment (15 months versus 15 months; *p* = 0.12) or overall survival (16 months versus 13 months; *p* = 0.10) for patients treated on genotype-matched versus genotype-unmatched trials.

### Germline testing

Of the patients who were asked during consent about return of incidental pathogenic germline mutations, 658/698 (94.3 %) indicated that they wished to receive these results. Two patients were identified with *TP53* variants in DNA extracted from blood. The first patient was a 36-year-old woman diagnosed with metastatic breast cancer, with a prior papillary thyroid cancer at the age of 28 years, who had a heterozygous germline *TP53* c.817C > T (p.Arg273Cys) pathogenic mutation. Her family history was notable for her mother who died from cancer of unknown primary at the age of 63 years and a maternal aunt with breast cancer at the age of 62 years. The second patient, a 77-year-old woman diagnosed with metastatic cholangiocarcinoma, had no family history of malignancy. We detected a heterozygous *TP53* c.524G > A (p.Arg175His) pathogenic mutation at 15 % allele frequency in the blood that was not present in tumor. This finding is not consistent with inherited Li-Fraumeni syndrome (LFS), but may represent either clonal mosaicism or an age-related or treatment-related mutation limited to blood.

## Discussion

We demonstrated that molecular profiling with mass-spectrometry-based genotyping or targeted NGS can be implemented in a large academic cancer center to identify patients with advanced solid tumors who are candidates for genotype-matched clinical trials. The rapid enrolment to our study reflects the high level of motivation of patients and their oncologists to pursue genomic testing that has been previously reported by our group [9, 10] and others [1, 11–13]. Disappointingly, only 5 % of patients who underwent successful molecular profiling in our study were subsequently treated on genotype-matched clinical trials, consistent with other centers. For comparison, the MD Anderson institutional genomic testing protocol matched 83/2000 (4 %) of patients [1], the SAFIR-01 breast cancer trial matched 28/423 (7 %) [14], and the British Columbia Cancer Agency Personalized Oncogenomics Trial matched 1/100 (1 %) [15]. To facilitate trial accrual, we incorporated multidisciplinary tumor board discussions, physician-directed email alerts with genotype-matched trial listings available at our institution, and individual physician summaries of profiling results. In spite of these efforts, the rate of genotype-matched clinical trial enrolment was low, due to patient deterioration, lack of available clinical trials, and unwillingness of patients to travel for clinical trial participation. There was no difference in proportion of patients treated on genotype-matched trials who underwent profiling using MALDI-TOF or a larger targeted NGS panel. This highlights how few somatic mutations are truly “druggable” through clinical trial matching, even in a large academic cancer center with a broad portfolio of phase I/II trials.

A key finding of our study is that patients in genotype-matched trials were more likely to achieve response than patients in genotype-unmatched trials. Albeit a non-randomized comparison, this finding comprises an important metric and distinguishes our molecular profiling program from other prospective studies that have not tracked longitudinal clinical outcome [1, 16, 17]. Von Hoff and colleagues were the first to report clinical outcome from a prospective molecular profiling (MP) study, with 18/66 (27 %) of patients who received treatment guided by MP data, including RNA-expression profiling and immunohistochemistry (IHC) or fluorescence in situ hybridization (FISH) testing for 11 markers, achieving a progression-free survival (PFS) ratio on MP-selected therapy/PFS on prior therapy) of ≥ 1.3 [18]. This study was performed prior to the era of multiplex mutation testing and many patients received MP-guided therapy with cytotoxic therapy using biomarker data that has not been shown to influence treatment response. An analysis of 1114 patients treated on investigational clinical trials at the Clinical Center for Targeted Therapy at MD Anderson Cancer Center reported that the response rate for patients with ≥1 molecular alteration treated on trials with matched therapy was higher (27 % versus 5 %, *p* < 0.0001) and the time to treatment failure was longer (5.2 versus 3.1 months; *p* < 0.0001) than those who received non-matched therapy [19]. Limitations of this study were that some patients underwent molecular testing after trial assignment and different sequential molecular tests such as polymerase chain reaction-based sequencing, IHC, and FISH, were performed based upon the patient’s tumor type.

The same investigators from MD Anderson recently reported the results of their prospective genomic profiling study that enrolled 500 patients with advanced refractory solid tumors assessed in their phase I program [20]. They utilized the FoundationOne™ 236-gene targeted sequencing panel and standard of care biomarker test results (such as ER, PR, and HER2 IHC for breast cancer) to inform treatment selection for commercially available therapies and clinical trial enrollment. A numerically higher rate of prolonged disease control (complete response, partial response, or stable disease ≥ 6 months) was observed in patients who received matched therapy (122/500) compared with those who received unmatched therapy (66/500) (19 % versus 8 %, *p* = 0.061). Higher matching scores, calculated based on the number of drug matches and genomic aberrations per patient, were independently associated with a greater frequency of prolonged disease control (22 % [high scores] versus 9 % [low scores], *p* = 0.024), longer time-to-treatment failure (hazard ratio [HR] = 0.52, 95 % confidence interval [CI] = 0.36–0.74, *p* = 0.0003), and survival (HR = 0.65, CI = 0.43–1.0, *p* = 0.05). Likewise, a retrospective review of 347 consecutive patients with advanced solid malignancies treated at the UC San Diego Moores Cancer Center who had targeted sequencing of archival tumor tissue using an earlier version of Foundation One™ (182-gene panel) reported a higher rate of disease control ≥ 6 months (34.5 %) for patients (87/342) treated with matched therapy compared with patients (93/342) treated with unmatched therapy [21]. In both of these studies, the rate of treatment matching (25 %) was significantly greater than our study (5 %). This may be due to the use of larger gene panels that include copy number alterations and recurrent translocations that may identify more “druggable” alterations for matched therapy; analysis of patient outcomes beyond therapeutic clinical trials that included off-label treatment matching; and varying definitions of genomic alteration and treatment-matching pairs. For instance, the UC San Diego Moores matched therapy cohort included 11 patients (13 %) with breast cancer who received endocrine therapy based on ER expression and 11 patients (13 %) with breast cancer who received HER2-directed therapy based on *ERBB2* (HER2) amplification. Since ER and HER2 testing are routinely performed in breast cancer patients to guide standard therapies, these patients would not have been included in our matched therapy cohort if the ER and HER2 status were known prior to enrollment in our molecular profiling study.

The only randomized trial that has prospectively assessed the utility of molecular profiling (SHIVA) reported no difference in objective response or PFS for patients treated with genotype-matched versus standard treatments [13]. More than 40 % of patients randomized in the SHIVA trial did not have genomic alterations identified and were included based upon expression of hormone receptors. Patients were matched to a limited range of approved targeted agents following a predefined algorithm that did not include best-in-class investigational agents that are being tested in early phase clinical trials. Despite the negative results of SHIVA, enthusiasm to conduct genomic-based clinical trials such as NCI-MATCH [12] [NCT02465060], and LUNG-MAP [22] [NCT02154490] remains strong to further define the value of precision medicine. The findings of our study, in which the majority of patients treated on genotype-matched trials were enrolled in phase I targeted therapy trials, are consistent with a recent meta-analysis of phase I trials that demonstrated a higher overall response rate (30.6 % versus 4.9 %, *p* < 0.001) and median PFS (5.7 months versus 2.95 months, *p* < 0.001) for targeted therapy trials that used biomarker-selection compared with those that did not [23].

Measuring the clinical utility of molecular profiling is difficult [3]. We did not comprehensively capture how testing results influenced clinical decisions outside of therapeutic clinical trial enrolment, such as reclassification of tumor subtype and site of primary based on mutation results. For example, we enrolled a patient with an unknown primary cancer with intra-abdominal metastases that was found to harbor a somatic *IDH1* p.Arg132Cys variant, leading to the reclassification as a likely intrahepatic cholangiocarcinoma. We also did not fully evaluate the use of testing results to avoid ineffective standard treatments (i.e. *KRAS* exon 4 somatic variants in colorectal cancer to inform decision not to use EGFR monoclonal antibody treatment) and treatment with approved targeted agents outside of their approved indications. Few patients in our study received targeted treatments based upon profiling results outside of clinical trials, due to limited access to targeted drugs outside of publicly funded standard-of-care indications in Ontario.

New technological advances are being studied in molecular profiling programs—including larger gene panels [2, 17]; whole exome [16], whole genome (WGS) or RNA sequencing (RNA-Seq) [24, 25]; and integrative systems biology analyses of deregulated cellular pathways [26]. Greater access to clinical trials for genomically characterized patients, such as umbrella and basket trial designs [27], may also improve the success of genotype-treatment matching. To assess whether decision support tools integrated at the point of care can improve enrollment of patients on genotype-matched trials, we are piloting a smart phone application to help physicians identify genotype-matched trials for their patients with profiling data.

There are several limitations of our study. Only a single archival sample was profiled for each patient, often obtained many years prior to molecular testing. Fresh biopsy of a current metastatic lesion for molecular profiling at the time of study enrolment may have yielded different results due to clonal evolution or tumor heterogeneity [28]. Our genomic testing was limited to hotspot point mutation testing or limited targeted sequencing and did not include gene copy number alterations or recurrent translocations that may be important for the selection of genotype-matched therapy. There were patients identified with potentially “druggable” mutations who were candidates for genotype-matched trials; however, they could not be enrolled because of the constraints of slot allocation in early phase clinical trials across multiple institutions or were deemed ineligible due to trial-specific exclusion criteria. Our study population also included many patients with heavily pre-treated metastatic disease who were not well enough for further therapy when results of molecular testing were reported. In addition, tumor response is an imperfect surrogate endpoint to assess therapeutic benefit in early phase clinical trials that should interpreted with caution [28]. We did not observe a difference in time on treatment or overall survival for patients treated on genotype-matched versus genotype-unmatched clinical trials. PFS data were not available in our cohort precluding a comparison of the outcome of genotype-matched therapy with the immediate prior line of treatment, as has been reported by other investigators [13, 14, 21].

## Conclusions

We provide preliminary evidence that genotype-matched trial treatment selected on the basis of molecular profiling was associated with increased tumor shrinkage, although only a small proportion of profiled patients benefitted from this approach. Through this initiative, we have created a valuable repository of data and tumor samples that are amenable to additional research and data sharing initiatives. Greater efforts should be made to expand opportunities for genotype-trial matching and further studies are needed to evaluate the clinical utility of targeted NGS profiling.

## Additional file

Additional file 1: Supplementary methods and **Tables S1–S4**. (DOCX 148 kb)

## Abbreviations

ASCP: AmpliSeq Cancer Panel
COMPACT: Community Oncology in Molecular Profiling Trial
FFPE: Formalin-fixed paraffin-embedded tissue
GEE: Generalized estimating equation
GENIE: Genomics, Evidence, Neoplasia, Information, Exchange
IHC: Immunohistochemistry
IMPACT: Integrated Molecular Profiling in Advanced Cancers Trial
MALDI-TOF: Matrix-assisted laser desorption/ionization time-of-flight
NGS: Next generation sequencing
PM: Princess Margaret Cancer Centre
PTEN: Phosphatase and tensin holog
RECIST: Response Evaluation Criteria in Solid Tumors
TSACP: TruSeq Amplicon Cancer Panel
WGS: Whole genome sequencing
