# Supplementary material for: Molecular profiling of advanced solid tumors and patient outcomes with genotype-matched clinical trials: the Princess Margaret IMPACT/COMPACT trial
Source: Genome Med. 2016 Oct 25;8:109. doi: 10.1186/s13073-016-0364-2 (PMC5078968; doi:10.1186/s13073-016-0364-2)
Supplement: Additional file 1: — Supplementary methods and Tables S1–S4. (DOCX 148 kb) [file 13073_2016_364_MOESM1_ESM.docx]

**Molecular Profiling Assays**

From March 2012 to November 2014, samples with >240ng DNA were tested by a

custom multiplex genotyping panel on a matrix-assisted laser desorption/ionization time- of-flight (MALDI-TOF) mass-spectrometry platform (MassARRAY, Agena Bioscience, San Diego, CA) to genotype 279 mutations within 23 genes. From April 2013 onward,

samples with >250ng DNA of acceptable quality (ΔCt< 3.0 assessed by FFPE QC Kit,

Illumina, San Diego, CA) were tested by TruSeq Amplicon Cancer Panel (TSACP, Illumina) on the MiSeq sequencer (Illumina) covering regions of 48 genes. Sequence alignment and base calling used MiSeq Reporter (Illumina), followed by variant calling using NextGENe v.2.3.1 software (SoftGenetics, State College, PA) and data review using the Integrative Genomics Viewer (IGV, Broad Institute). From November 2014 onward, samples of >10 ng DNA which did not meet criteria as defined for TSACP were tested by Ion AmpliSeq Cancer Panel (ASCP, ThermoFisher Scientific) on the Ion Proton sequencer (ThermoFisher Scientific) covering regions of 50 genes. Sequence alignment and base calling was performed by Torrent Suite software (ThermoFisher Scientific) and analysis using NextGENe v.2.3.1 and IGV software. All NGS analyses used hg19, NCBI Build 37, as reference genome.

Somatic variants identified met laboratory-defined thresholds of >500x read coverage and allele frequency of >10%. Recurrent mutations between 400-500X coverage or 5-

10% allele fraction were reported if they were verified by an orthogonal molecular method. Three genes with read depth consistently falling below 500x on TSACP (*GNAS*, *HRAS*, *CDKN2A*) were not included in the data analysis for this manuscript. Testing of germline DNA extracted from peripheral blood lymphocytes was performed on all cases using the same NGS platform to differentiate somatic and germline variants.

**Table S1.** 279 variants in 23 genes detected by the custom MALDI-TOF MS assay (MassArray, Agena Biosciences).

| **Gene** | **Variant (amino acid change)** | **Gene** | **Variant (amino acid change)** | **Gene** | **Variant (amino acid change)** |
| --- | --- | --- | --- | --- | --- |
| *AKT1* | E17K | *FGFR2* | S252W, Y375C, N549K | *NOTCH1* | L1586P, F1593S |
| *AKT2* | E17K, S302G, R371H | *FGFR3* | R248C, S249C, G370C, |  | L1594P, R1599P, L1601P |
| *AKT3* | E17K |  | S371C, Y373C, A391E |  | I1617N, L1679P, Q2460* |
| *BRAF* | G464R, G464V/E, G466R, F468C, |  | K650Q/E, K650T/M, G697C | *NRAS* | G12V/A/D, G12C/R/S |
|  | G469S/E/A/V/R, D594V/G, |  |  |  | G13V/A/D, G13C/R/S, A18T, |
|  | F595L, G596R, L597S/R/Q/V/P, T599I, | *HRAS* | G12R/S, G12V/A/D, G13C/R/S, |  | Q61L/R/P, Q61H, Q61E/K |
|  | V600E/K/R/L/D/G/M, K601N, K601E |  | Q61H, Q61L/R/P, Q61K | *PDGFRA* | V561D, S566_E571delinsK, T674I, F808L |
| *CDK4* | R24C, R24H | *KIT* | D52N, Y503_F504insAY |  | D842V, D842Y, D842_H845del |
| *CTNNB1* | A13T, A21T, V22A, D32Y/N/H, |  | K550_K558del, W557R/G |  | I843_S847delinsT, I843_D846del |
|  | D32G/A/V, S33C/F/Y, S33P/A, |  | K558_V560del, K558_E562del, |  | D846Y, N870S, D1071N |
|  | G34E/V, G34R, S37A/P, S37C/F/Y, |  | V559I, V559D/A/G, V559del, | *PIK3CA* | R38H, R88Q, N345K, |
|  | T41A/P/S, T41I, S45C/F/Y, S45P/A |  | V559_V560del, V560del, V560D/G, |  | C420R, P539R, E542K/Q |
| *EGFR* | R108K, T263P, A289V, G598V, |  | E561K, Y570_L576del, L576P, D579del, |  | E545K/Q, E545A/V/G |
|  | E709K/Q, E709A/G/V, G719S/C/A, |  | K642E, V654A, D816V, D816H/Y, D816E |  | Q546K/E, H701P, C901F |
|  | E746_A750del, E746_T751del, |  | D820Y/H, D820A/G, N822H/Y, N822I |  | Y1021C, T1025A, M1043I |
|  | L747_T751del, L747_S752del, A750P, |  | N822K, Y823D, V825A, A829P, E839K |  | H1047R/L, H1047Y, G1049R/S |
|  | T751A/I, S768I, D770_N771insG, | *KRAS* | G12V/A/D/C/S/R/F, G13V/D | *RET* | C634R, C634Y, C634W |
|  | D770E,T790M, T854A, L858R, L861Q |  | A59T, Q61E/K, Q61L/R/P, Q61H |  | E632_L633del, A664D, M918T |
| *ERBB2* | L755S, L755P, D769H, G776S, G776LC, | *MEK1* | Q56P, P124L | *SMO* | T640A |
|  | A775_G776insYVMA, G776VC, V777L, | *MET* | H1112Y, H1112R/L, Y1248C, Y1248H | *STK11* | Q37*, Q170*, D194Y, D194N |
|  | S779_P780insVGS, P780_Y781insGSP |  | Y1253D, M1268T |  | D194V, G196V, E199K/Q/* |
| FGFR1 | G70R, S125L, T141R, P252T, V664L |  |  |  | P281L, W332* |

**Table S2.** 48 genes included in the TruSeq Amplicon Cancer Panel (TSACP, Illumina), which includes 212 amplicons covering a total genomic region 35.84 kb.

| *ABL1* | *AKT1* | *ALK* | *APC* |
| --- | --- | --- | --- |
| *ATM* | *BRAF* | *CDH1* | *CDKN2A* |
| *CSF1R* | *CTNNB1* | *EGFR* | *ERBB2* |
| *ERBB4* | *FBXW7* | *FGFR1* | *FGFR2* |
| *FGFR3* | *FLT3* | *GNA11* | *GNAS* |
| *GNAQ* | *HNF1A* | *HRAS* | *IDH1* |
| *JAK2* | *JAK3* | *KDR* | *KIT* |
| *KRAS* | *MET* | *MLH1* | *MPL* |
| *NOTCH1* | *NPM1* | *NRAS* | *PDGFRA* |
| *PIK3CA* | *PTEN* | *PTPN11* | *RB1* |
| *RET* | *SMAD4* | *SMARCB1* | *SMO* |
| *SRC* | *STK11* | *TP53* | *VHL* |

**Table S3.** 50 genes included in the Ion AmpliSeq Cancer Panel v2 (ASCP, ThermoFisher), which includes 207 amplicons covering a total genomic region of 22 kb.

| *ABL1* | *AKT1* | *ALK* | *APC* |
| --- | --- | --- | --- |
| *ATM* | *BRAF* | *CDH1* | *CDKN2A* |
| *CSF1R* | *CTNNB1* | *EGFR* | *ERBB2* |
| *ERBB4* | *EZH2* | *FBXW7* | *FGFR1* |
| *FGFR2* | *FGFR3* | *FLT3* | *GNA11* |
| *GNAS* | *GNAQ* | *HNF1A* | *HRAS* |
| *IDH1* | *IDH2* | *JAK2* | *JAK3* |
| *KDR* | *KIT* | *KRAS* | *MET* |
| *MLH1* | *MPL* | *NOTCH1* | *NPM1* |
| *NRAS* | *PDGFRA* | *PIK3CA* | *PTEN* |
| *PTPN11* | *RB1* | *RET* | *SMAD4* |
| *SMARCB1* | *SMO* | *SRC* | *STK11* |
| *TP53* | *VHL* |  |  |

**Table S4.** Factors associated with higher overall response rate by RECIST in therapeutic clinical trials.

|  | **p-value** |
| --- | --- |
| Trial matching by genotype (genotype-matched vs genotype-unmatched) | 0.021 |
| Gender (female vs male) | 0.034 |
| Trial Phase (I vs II/III) | 0.058 |
| Investigational Agent Class (Targeted Monotherapy vs other) | 0.067 |
| Age (≤58 vs >58) | 0.217 |
| Prior lines of systemic therapy (<3 vs ≥3) | 0.391 |
| Tumor Type (Gynecological vs other) | 0.717 |
| Genotyping Platform (MALDI-TOF MS vs other) | 0.867 |
